# Intracellular K^+^-Responsive Block Copolymer Micelles for Targeted Drug Delivery of Curcumin

**DOI:** 10.3389/fbioe.2022.919189

**Published:** 2022-06-30

**Authors:** Mingyue Jiang, Le Chen, Bo Chen, Qinghua Yu, Xianming Zhang, Weihong Jing, Limei Ma, Tao Deng, Zhangyou Yang, Chao Yu

**Affiliations:** ^1^ Chongqing Key Laboratory for Pharmaceutical Metabolism Research, College of Pharmacy, Chongqing Medical University, Chongqing, China; ^2^ Research Center of Pharmaceutical Preparations and Nanomedicine, College of Pharmacy, Chongqing Medical University, Chongqing, China; ^3^ Chongqing Pharmacodynamic Evaluation Engineering Technology Research Center, College of Pharmacy, Chongqing Medical University, Chongqing, China

**Keywords:** curcumin, polymer micelles, K^+^ -triggered drug release, responsive host–guest system, anticancer

## Abstract

Curcumin (CUR) is a natural bioactive compound that has attracted attention as a “golden molecule” due to its therapeutic properties against several types of tumors. Nonetheless, the antitumor application of CUR is hampered due to its extremely low aqueous solubility and chemical instability. Herein, a novel type of CUR-loaded polymeric micelles with intracellular K^+^-responsive controlled-release properties is designed and developed. The polymeric micelles are self-assembled by poly (*N*-isopropylacrylamide-*co*-acryloylamidobenzo-15-crown-5-*co*-*N*, *N*-dimethylacrylamide)-b-DSPE (PNDB-b-DSPE) block copolymers, and CUR. CUR is successfully loaded into the micelles with a CUR loading content of 6.26 wt%. The proposed CUR-PNDB-DSPE polymeric micelles exhibit a significant CUR release in simulated intracellular fluid due to the formation of 2 : 1 ‘‘sandwich’’ host–guest complexes of 15-crown-5 and K^+^, which lead to the hydrophilic outer shell of micelles to collapse and the drug to rapidly migrate out of the micelles. *In vitro*, the B16F10 cell experiment indicates that CUR-PNDB-DSPE micelles exhibit a high cellular uptake and excellent intracellular drug release in response to the intracellular K^+^ concentration. Moreover, CUR-PNDB-DSPE micelles show high cytotoxicity to B16F10 cells compared to free CUR and CUR-PEG-DSPE micelles. The polymeric micelles with intracellular K^+^-responsive controlled release properties proposed in this study provide a new strategy for designing novel targeted drug delivery systems for CUR delivery for cancer treatment.

## Introduction

Curcumin (CUR) is a natural bioactive compound found in turmeric that has attracted attention as a “golden molecule” due to its therapeutic properties against several types of tumors (Sharma et al., 2005; Fan et al., 2013; Zoi et al., 2021). Nonetheless, the antitumor application of CUR is hampered because of its extremely low aqueous solubility and chemical instability, resulting in low bioavailability and fast metabolism (Siviero et al., 2015; Nelson et al., 2017). Nano-based drug delivery systems have been exploited to improve CUR solubility, protect CUR against hydrolysis and enzymatic reduction, and facilitate targeted accumulation in tumor tissues (Naksuriya et al., 2014; D'Angelo et al., 2021; Tu et al., 2022). Polymeric micelles, able to encapsulate hydrophobic drugs in the micelle core, have gathered significant interest as nano-based drug delivery systems to deliver CUR because of their good biocompatibility, prolonged blood circulation, and modified release pattern (Yadav et al., 2020; Machtakova et al., 2022). Moreover, to achieve site-specific release at targeting regions, stimuli-responsive polymeric micelles have been designed to respond to environmental stimuli such as tumor extracellular and/or intracellular microenvironment (Wang et al., 2016; Cabral et al., 2018; Shao et al., 2020; Shi et al., 2022). Particularly, stimuli-responsive polymeric micelles that could achieve controlled intracellular release of drugs exert significant therapeutic effects for reducing the leakage of the drug in the systemic circulation and maximizing the drug release to the targeted tumor cells. Potassium ion (K^+^) plays an important role in biological systems, where the normal serum K^+^ level in the human body is in the range of 3.5–5.5 mM, while the value of the intracellular K^+^ concentration is about 30 times that of the extracellular K^+^ concentration (Kuo et al., 2001; Armstrong, 2003). Therefore, the design and preparation of stimuli-responsive polymeric micelles that can recognize the signal of intracellular K^+^ concentration for controlled intracellular release of CUR are of great significance in cancer treatment.

To date, a lot of investigations have been carried out on stimuli-responsive polymeric micelles loaded with CUR for cancer treatment, in which most polymeric micelles are designed in response to endogenous stimuli including variations in pH (Yu et al., 2014; Cai et al., 2016), redox potential (Li et al., 2018; Zhao et al., 2020), and enzyme concentration (Li et al., 2017). For instance, polymeric micelles designed to respond to pH changes, acidic interstitial pH (pH 6.5–7.2), or endosomal/lysosomal pH (pH 6.5–4.5) are usually equipped with ionizable groups or pH-cleavable linkages and can release CUR intratumorally or intracellularly (Bae et al., 2003; Mura et al., 2013). However, it is difficult to achieve satisfactory pH-responsive release in response to such subtle pH variation. Redox-sensitive polymeric micelles can be modified with disulfide bonds for drug conjugation or cross-linking, which are cleaved by elevated glutathione in the cytosol (2–10 mM), which is 1000-fold higher than the levels at the extracellular fluid (Kuppusamy et al., 2002; Lopez-Mirabal and Winther, 2008). Moreover, enzyme-sensitive micelles can be prepared by introducing moieties in the building segments which are selectively recognized and degraded by enzymes overexpressed in interstitial or intracellular environments (Andresen et al., 2010). Nevertheless, the core-shell structure of polymeric micelles may limit the access of bulky enzymes to the enzyme-cleavable moieties within the core, thus reducing the drug release rate. In addition, as aforementioned, there is an obvious variation in K^+^ concentration between extracellular (3.5–5.5 mM) and intracellular fluid (140–150 mM). However, until now, K^+^-responsive polymeric micelles encapsulating CUR with controlled-release behaviors have not been reported yet. It has been reported that crown ether 15-crown-5 can selectively recognize and capture K^+^ by forming stable 2 : 1 ‘‘sandwich-type’’ host–guest complexes (Yu et al., 2013; Jiang et al., 2014; Jiang et al., 2016). K^+^-responsive micelles self-assembled by poly (ethylene glycol)-b-poly (*N*-isopropylacry-lamide-*co*-benzo-18-crown-6-acrylamide) (PEG-b-P(NIPAM-*co*-B18C6Am)) block copolymers have been reported to load prednisolone acetate (Yan et al., 2017). However, the therapeutic properties of the micelles are not studied, and these micelles should be stored in solution at 45 °C, which limits their further use. Therefore, the design and preparation of the K^+^-responsive micelles loaded with CUR that can recognize intracellular K^+^ signals and achieve adjustable controlled release characteristics are of great importance for treating cancer.

**SCHEME 1:**
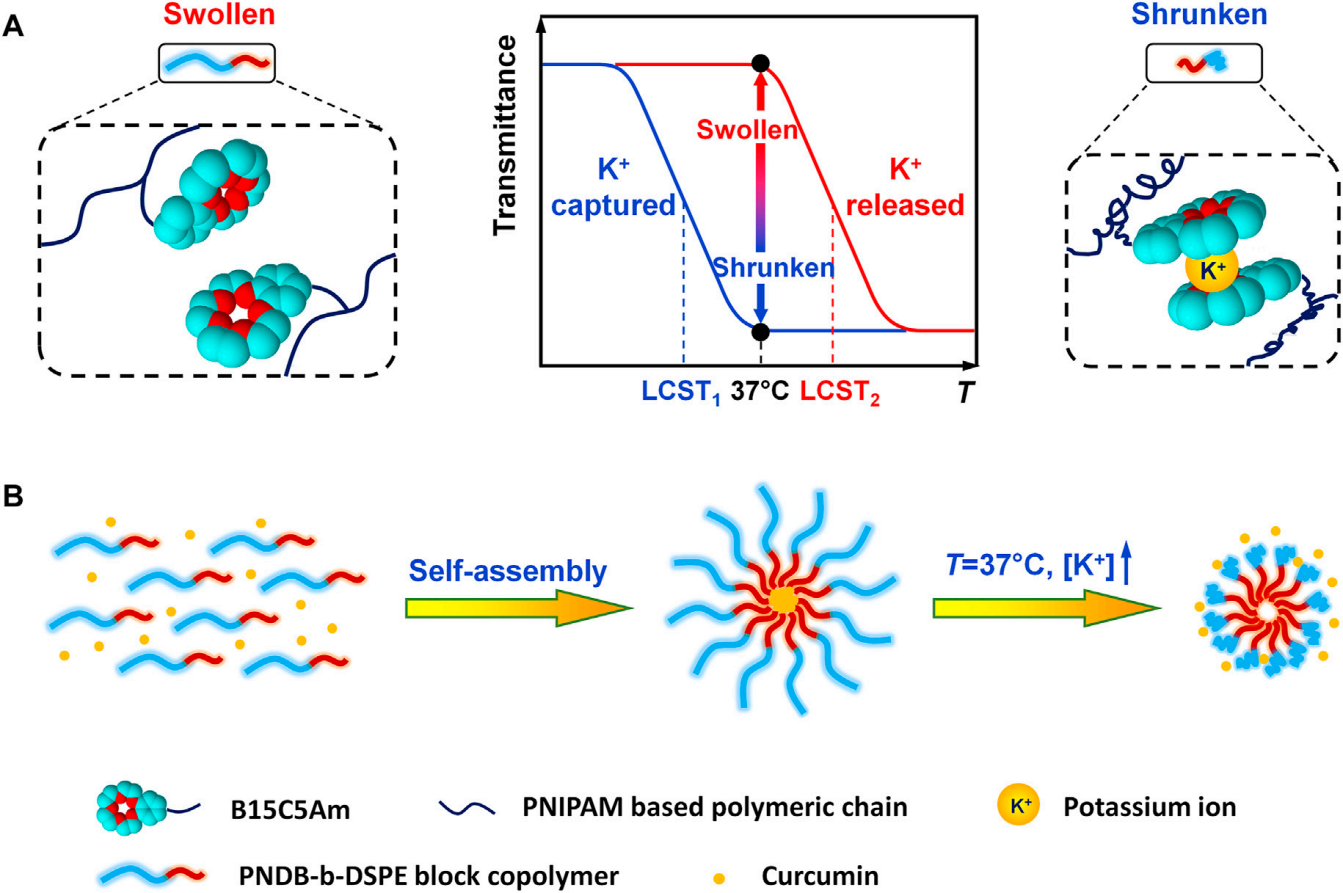
Schematic illustration of K^+^-responsive behavior of CUR-PNDB-DSPE micelles. **(A)** Different states of block copolymers responding to K^+^. **(B)** Self-assembly of block copolymers to form micelles and K^+^-responsive drug release of micelles.

In this study, we develop a novel type of CUR-loaded polymeric micelles with intracellular K^+^-responsive drug-release properties. Poly (*N*-isopropylacrylamide-*co*-acryloylamidobenzo-15-crown-5-*co*-*N*, *N*-dimethylacrylamide) (PNDB) copolymers with 15-crown-5 as the K^+^ sensor are used as the hydrophilic block, which presents an obvious K^+^-responsive hydrophilic/hydrophobic phase transition. Inspired by *N*-(carbonyl-methoxypolyethylene glycol)-1,2-distearoyl-sn-glycero-3-phosphoethanolamine (mPEG-DSPE), which has been approved by the FDA for medical applications, DSPE is used as the hydrophobic block. As illustrated in Scheme 1, when the environmental K^+^ concentration is low, PNDB copolymers present a hydrophilic and swollen state at normal physiological temperature (37°C). The micelles are self-assembled by PNDB-b-DSPE block copolymers and CUR. The hydrophobic DSPE segments in the copolymers form the micellar core that acts as a reservoir for CUR, while the hydrophilic and swollen PNDB copolymers form the micellar shell that can maintain colloidal stability. After isothermally transferring into the intracellular fluid, where the environmental K^+^ concentration is significantly increased, the adjacent 15-crown-5 receptors capture K^+^ to form stable 2 : 1 ‘‘sandwich-type’’ host–guest complexes, which would disrupt the hydrogen bonding between the oxygen atoms in 15-crown-5 and the hydrogen atoms of water, resulting in the hydrophobic and shrunken state of PNDB copolymers. Therefore, the hydrophilic outer shell collapses, and the drug can migrate out of the micelles in intracellular fluid, and thus micelles exhibit a fast release of CUR. CUR-PNDB-DSPE micelles demonstrate an obvious cellular uptake and excellent intracellular drug release in the B16F10 cell study. Moreover, CUR-PNDB-DSPE micelles show high cytotoxicity to B16F10 cells compared to that of free CUR and CUR-PEG-DSPE micelles. The results in this study provide valuable guidance for designing novel targeted drug delivery systems serving as a delivery vehicle for CUR with intracellular K^+^-responsive controlled release properties.

## Materials and Methods

### Materials

*N*-isopropylacrylamide (NIPAM, purchased from Rhawn) is purified by recrystallization with a hexane/acetone mixture (v/v, 50/50). Benzo-15-crown-5-acrylamide (B15C5Am) is synthesized from 4′-nitro-benzo-15-crown-5 (NB15C5, TCI) according to previously reported procedures (Mi et al., 2008; Mi et al., 2010). *N*, *N*-dimethylacrylamide (DMAm, Sigma-Aldrich) is passed through a previously washed prepacked column of inhibitor removers (Aldrich). 2,2′-azoisobutyronitrile (AIBN, Aladdin) is used after recrystallization with ethanol. 2-(dodecylthiocarbonothioylthio)-2-methylpropanoic acid (DDMAT, Adamas), 1, 2-distearoyl-sn-glycero-3-phosphoethanolamine (DSPE, Aladdin), 1-(3-dimethylaminopropyl)-3-ethyl carbodiimide hydrochloride (EDC, Rhawn), *N*-hydroxysuccinimide (NHS, Rhawn), curcumin (Macklin), and mPEG-DSPE (2000Da, Macklin) are used without further purification. All solvents and other chemicals are of analytical grade and used as received. Deionized water (18.2 MΩ, 25°C) from a water purification system (Research Water Purification Technology Co., Ltd, China) is used throughout this study.

### Synthesis of Block Copolymers

The proposed PNDB-b-DSPE copolymers are prepared by a two-step reaction method combining the reversible addition-fragmentation chain transfer polymerization (RAFT) method and condensation reaction. The detailed synthesis route is illustrated in Supplementary Scheme S1. First, carboxyl-terminated PNDB copolymers are prepared by thermally initiated RAFT of NIPAM, B15C5Am, and DMAm comonomers in 1,4-dioxane with DDMAT as the transfer agent and AIBN as the initiator. The concentration of total monomers (NIPAM, B15C5Am, and DMAm) is 0.3 mol·L^−1^, and the molar ratios of B15C5Am, AIBN, and DDMAT to the total comonomers are kept constant at 20 mol%, 1 mol%, and 0.8 mol%, respectively. The reaction solution is bubbled with N_2_ gas for 20 min to remove dissolved oxygen and then is heated to 70 °C to initiate the copolymerization. The copolymerization is carried out at 70 °C for 4 h under an N_2_ atmosphere. The obtained PNDB copolymers solution is diluted with tetrahydrofuran and purified three times by reprecipitation with excess of methyl tert-butyl ether from tetrahydrofuran to thoroughly remove the unreacted monomers and impurities and then dried under vacuum. The lower critical solution temperature (LCST) of copolymers can be flexibly controlled by adjusting the molar ratios of the DMAm monomer during the synthesis process, and the theoretical feeding molar ratios of DMAm to the total monomers are designed as 50, 60, and 70 mol%, which are labeled as PNDB1, PNDB2, and PNDB3, respectively.

Next, PNDB-b-DSPE block copolymers are synthesized by the condensation reaction of carboxyl-terminated PNDB and DSPE by using EDC and NHS as dehydration catalysts. Briefly, the dispersion of carboxyl-terminated PNDB copolymers in chloroform that mixed with EDC and NHS is stirred below 4°C for 20 min under an N_2_ atmosphere. DSPE dissolved in chloroform with a small amount of triethylamine is then added dropwise to the mixed solution, and the reaction is carried out for 24 h. Then, the synthesized copolymers are purified by dialysis (cutoff 1,000 Da) against deionized water. The prepared PNDB-b-DSPE block copolymers are obtained by freeze-drying. Three kinds of PNDB-b-DSPE block copolymers with different mole ratios of DMAm are synthesized and labeled as PNDB-b-DSPE1, PNDB-b-DSPE2, and PNDB-b-DSPE3.

### Compositional Characterizations of Block Copolymers

The chemical compositions of carboxyl-terminated PNDB and PNDB-b-DSPE copolymers are confirmed by Fourier transform infrared spectroscopy (FT-IR, Nicolet iS5, Thermo Fisher Scientific) by using the KBr disc technique. The compositions of the copolymers are determined by nuclear magnetic resonance spectrometry (^1^H NMR, Bruker, America). The weighted average molecular weight of PNDB-b-DSPE is determined by gel permeation chromatography (GPC, Waters-2410, Waters) using tetrahydrofuran as the mobile phase and polystyrene as the standard. The critical micelle concentration (CMC) values of PNDB-b-DSPE block copolymers are determined using a fluorescence spectrometer (RF-5301PC, Shimadzu), and pyrene is used as a fluorescent probe.

### Characterization of K^+^-Responsive Behaviors of Block Copolymers

The K^+^-responsive behaviors of carboxyl-terminated PNDB and PNDB-b-DSPE copolymers with different contents of DMAm units are comprehensively investigated by measuring their corresponding LCST values in aqueous solutions containing K^+^ ions with different concentrations. An aqueous solution with K^+^ concentration of 5 mM and Na^+^ concentration of 150 mM is regarded as the simulated extracellular fluid, and the aqueous solution with K^+^ concentration of 150 mM and Na^+^ concentration of 5 mM is regarded as the simulated intracellular fluid. The LCST values of the copolymers are evaluated by measuring the optical transmittance of the copolymer aqueous solutions at 500 nm as a function of temperature using a UV–Vis spectrophotometer (UV-2600, Shimadzu) equipped with a temperature-controlled cell (TCC-100, Shimadzu). The LCST value is defined as the temperature at which the optical transmittance decreases to half of the initial value. To minimize salting-out effects, nitrates are chosen as the model salts (Inomata et al., 1992; Zhang et al., 2005). The concentrations of the block copolymers in different aqueous solutions are all fixed at 0.5 wt%.

### Preparation of Micelles

CUR-loaded micelles are prepared by a thin-film hydration method. Briefly, PNDB-b-DSPE copolymers and CUR (mass ratio, 10:1) are dissolved in methanol. The organic solvent is removed by rotary evaporation at 37°C, and the thin film is hydrated with PBS at 37°C for 1 h. The micelle solution (CUR-PNDB-DSPE micelles) is filtered through a 0.22-μm syringe filter (Jinteng, China) to remove free curcumin, lyophilized, and stored at 4°C for further use. Blank micelles are synthesized by using the same procedure without encapsulation of CUR. In addition, CUR-loaded micelles self-assembled by mPEG-DSPE copolymers and CUR (CUR-PEG-DSPE micelles) served as the control group.

### Characterization of Micelles

The hydrodynamic diameters and zeta potentials of micelles are measured by dynamic light scattering (DLS, ZEN3690, Malvern) with highly diluted micelle dispersions in aqueous solutions. Moreover, the hydrodynamic diameters of CUR-PNDB-DSPE micelles in serum (10%, v/v) are monitored for 24 h. The size and morphology of micelles are observed by a transmission electron microscope (TEM, JEM-1200EX, JEOL). The micelles are diluted with distilled water and dropped on a copper grid covered with nitrocellulose. All specimens for TEM measurements are dried at room temperature and negatively stained with 2% phosphotungstic acid before observation. Encapsulation efficiency (EE) and loading content (LC) of CUR are detected by UV–Vis spectrophotometer (UV-2600, Shimadzu). EE is calculated by using the mass ratio of their loaded drug to the initially fed drug, while LC is calculated by using the mass ratio of their loaded drug to the drug-loaded micelles.

### Characterization of K^+^-Responsive Controlled-Release Behaviors of Micelles

*In vitro* drug release behavior of micelles is performed by dialysis in simulated extracellular fluid or simulated intracellular fluid with 0.5 wt% Tween 80. The molecular weight cutoff size of the dialysis membrane is 3,000 Da. Briefly, 5 ml of CUR-PNDB-DSPE micelles and CUR-PEG-DSPE micelle solution are placed into a dialysis bag, sealed on both ends, and immersed in the dissolution medium (50 ml) under 100 rpm stirring at 37°C. At the predetermined time intervals, 1 ml of the solution is withdrawn from the release medium and replaced by an equal volume of fresh medium. With suitable dilution, the concentrations of CUR in the release medium are determined by a UV–Vis spectrophotometer at 426 nm.

### Cell Culture

Metastatic murine melanoma cells (B16F10) are incubated in Dulbecco’s modified Eagle’s medium nutrient mixture F12 (DMEM/F12, Gibco) containing 10% fetal bovine serum (FBS, Bio-Channel) and 1% antibiotic (penicillin/streptomycin/amphotericin B, Beyotime) solution in a humidified atmosphere with 5% CO_2_ at 37°C.

### Cellular Uptake and Intracellular Drug Release

The cellular uptake and intracellular drug release of CUR-loaded micelles are evaluated by using a fluorescence microscope. B16F10 cells are seeded into a 24-well plate with 1 × 10^5^ cells per well on sterile coverslips and incubated for 24 h. Subsequently, the medium is replaced, and then the cells are incubated with free CUR, CUR-PNDB-DSPE micelles, or CUR-PEG-DSPE micelles, with the same concentration of CUR at 20 μg mL^−1^, for 1, 4, and 8 h, respectively. Free CUR solutions are prepared by using DMSO to improve the solubility, and the final concentration of DMSO in the culture medium is 0.2% (v/v). After incubation, the cells are washed with ice-cold PBS and fixed with 4% polyoxymethylene for 20 min. Then, the samples are washed with PBS three times followed by staining with DAPI in a dark environment. Finally, the prepared samples are observed using a laser scanning confocal microscope (CLSM, Leica, Germany).

### Cytotoxicity Assays

B16F10 cells are seeded in 96-well plates at a density of 1 × 10^4^ cells per well and cultured for 24 h. After that, the culture medium is replaced by free CUR, CUR-PNDB-DSPE micelles, and CUR-PEG-DSPE micelles at concentrations of CUR equivalents ranging from 0.078–20 μg mL^−1^. Blank PNDB-DSPE micelles without encapsulation of CUR are used as vehicle control, with concentrations of PNDB-DSPE copolymers ranging from 20–400 μg mL^−1^. The cells are incubated for 24 and 48 h. Afterward, in the dark, fresh culture medium (100 μL) containing CCK-8 solution (10 μL, 5 mg/ml in PBS) is added and incubated for 2 h. Then, the absorbance at 450 nm is measured by using a Varioskan LUX microplate reader (Thermo Fisher Scientific, United States). Cell viability is calculated according to the absorbance values.

## Results and Discussion

### Compositional Characterizations of Block Copolymers

**FIGURE 1:**
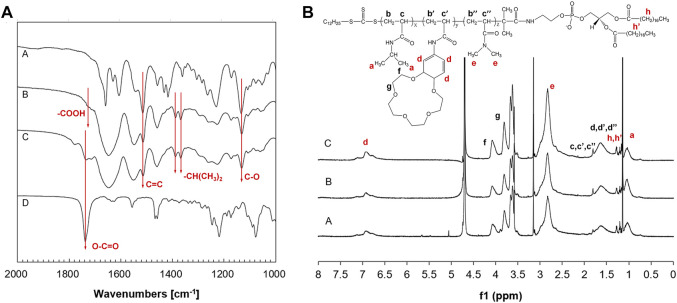
**(A)** FT-IR spectra of B15C5Am (Curve A), PNDB (Curve B), PNDB-b-DSPE (Curve C), and DSPE (Curve D). **(B)** 1H NMR spectra of PNDB-b-DSPE1 (Curve A), PNDB-b-DSPE2 (Curve B), and PNDB-b-DSPE3 (Curve C) block copolymers.

FT-IR spectra of carboxyl-terminated PNDB and PNDB-b-DSPE copolymers are shown in Figure 1A. From the FT-IR spectra, successful fabrications of carboxyl-terminated PNDB and PNDB-b-DSPE copolymers are confirmed. Specifically, the characteristic bands of a benzo-15-crown-5 group of B15C5Am (Curve A), including a strong peak at 1,513 cm^−1^ (shoulder peak) for C=C skeletal stretching vibration in the phenyl ring, a peak at 1,130 cm^−1^ for C−O asymmetric stretching vibration in R−O−R’, and double peaks at 1,386 cm^−1^ and 1,367 cm^−1^ for isopropyl group of NIPAM, are both found in the FT-IR spectra of carboxyl-terminated PNDB (Curve B) and PNDB-b-DSPE copolymers (Curve C). Furthermore, the weak characteristic peak at 1718 cm^−1^ for the carboxylic group in the FT-IR spectrum of carboxyl-terminated PNDB (Curve B) disappears in the FT-IR spectrum of PNDB-b-DSPE copolymers (Curve C), and the characteristic peak at 1737 cm^−1^ for the ester group of DSPE (Curve D) is found in the FT-IR spectrum of PNDB-b-DSPE copolymers (Curve C), which indicates the successful chemical modification of DSPE on the carboxyl-terminated PNDB.

^1^H NMR spectra are used to further confirm the chemical structure of the block copolymers as shown in Figure 1B. The ^1^H chemical shifts at around 1, 7, and 2.8 ppm are the characteristic peaks of protons on the isopropyl, benzene ring, and methyl groups, respectively. Calculated from the ^1^H NMR results, the molar ratio of DMAm to total monomers of PNDB1, PNDB2, and PNDB3 is 56.93, 60.23, and 65.58%, respectively, which is similar with corresponding theoretical feeding molar ratios. In addition, the molecular weights of copolymers are also measured by the GPC method shown in Supplementary Figure S1, and the number of average molecular weights (M_n_) of PNDB-b-DSPE1, PNDB-b-DSPE2, and PNDB-b-DSPE3 is 2,300, 2,510, and 2,550, respectively. Thus, mPEG-DSPE with molecular weights of 2000 is used to prepare micelles as a control group for further study.

### K^+^-Responsive Behaviors of Block Copolymers

The effects of different cations on the stability of micelles result from the hydrophilic–hydrophobic states of the block copolymers. The effects of K^+^, Na^+^, Mg^2+^, and Ca^2+^ ions, which are the main cations in the human body, on the phase transition behaviors of PNB copolymers have been systematically investigated in the reported study (Yu et al., 2013). The results indicate that PNB copolymers only show good selectivity and sensitivity toward K^+^ among those cations, which is mainly caused by the formation of stable 2:1 (ligand/ion) “sandwich-type” host–guest complexes between 15-crown-5 units and K^+^ ions, which leads to the contraction of copolymers. However, other cations cannot form stable 2:1 host–guest complexes with 15-crown-5 units. Therefore, only the effects of K^+^ concentrations on the LCST values of the PNDB-b-DSPE block copolymers are investigated.

**FIGURE 2:**
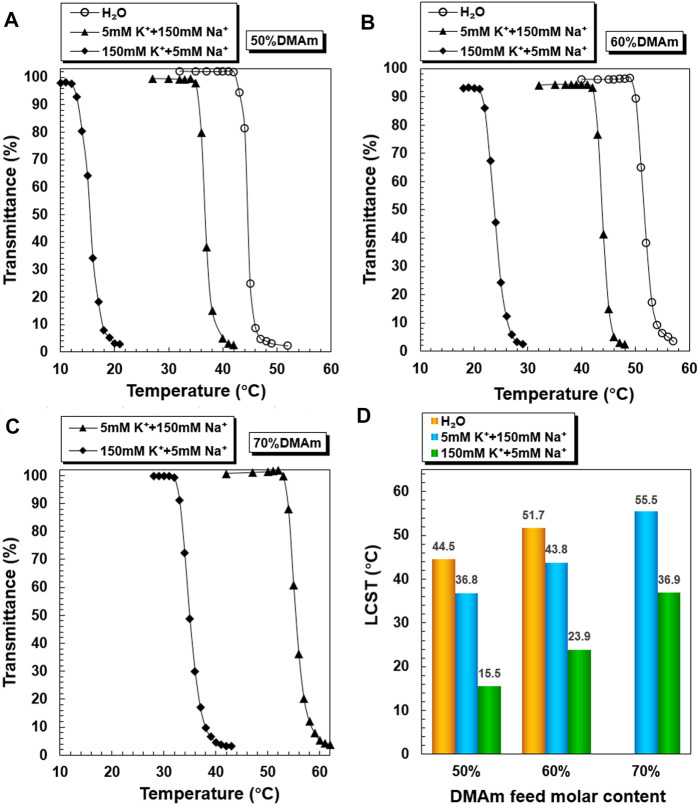
K^+^-responsive behaviors of PNDB copolymers. Temperature-dependent phase transition behaviors of PNDB1 **(A)**, PNDB2 **(B)**, and PNDB3 **(C)** copolymer solutions with different K^+^ concentration levels. LCST values of PNDB1, PNDB2, and PNDB3 copolymer solutions with different K^+^ concentration levels **(D)**.

The temperature-dependent transmittance changes of PNDB1, PNDB2, and PNDB3 copolymer solutions with different K^+^ concentrations are shown in Figures 2A–C. As expected, by increasing the temperature, PNDB1, PNDB2, and PNDB3 copolymer solutions with different K^+^ concentrations all undergo a rapid optical transmittance change when the environmental temperature varies across a corresponding temperature region due to the excellent thermoresponsive property of the PNIPAM chain, while with introducing crown ether 15-crown-5 units in the copolymers, the LCST of PNDB1, PNDB2, and PNDB3 copolymers exhibits an obvious negative shift in K^+^ solution compared with that of deionized water. Such a distinct change in the phase transition temperature results from the formation of stable 2:1 “sandwich” host–guest complexes of 15-crown-5 with K^+^, whereas the phase transition behaviors of PNDB3 copolymers with a 70% DMAm feeding ratio in water, with relatively high LCSTs, are not presented since it is beyond the detection limit of the instrument. With increasing K^+^ concentration from 5 to 150 mM, the negative shift degree of LCST is increased due to the formation of more 2:1 ‘‘sandwich’’ host–guest complexes. For PNDB1, PNDB2, and PNDB3, the LCST values are 36.8, 43.8, and 55.5°C in the simulated extracellular fluid and dramatically decrease to 15.5, 23.9, and 36.9°C, respectively, in the simulated intracellular fluid (Figure 2D). As indicated, the LCST values in simulated extracellular and intracellular fluids of PNDB copolymers increase with increasing molar ratio of DMAm in PNDB copolymers (Katsumoto et al., 2005). It is reported that, on the one hand, an increased molar ratio of DMAm in NIPAM-based copolymers leads to fewer hydrogen donors for intramolecular hydrogen bonding, and on the other hand, more thermal energy is required for dehydrating the C=O groups of DMAm and increasing the strength of intramolecular hydrogen bonding among neighboring units for DMAm. Thus, an increased molar ratio of DMAm could improve the hydrophilicity of the PNDB copolymers and increase the corresponding LCST values.

**FIGURE 3:**
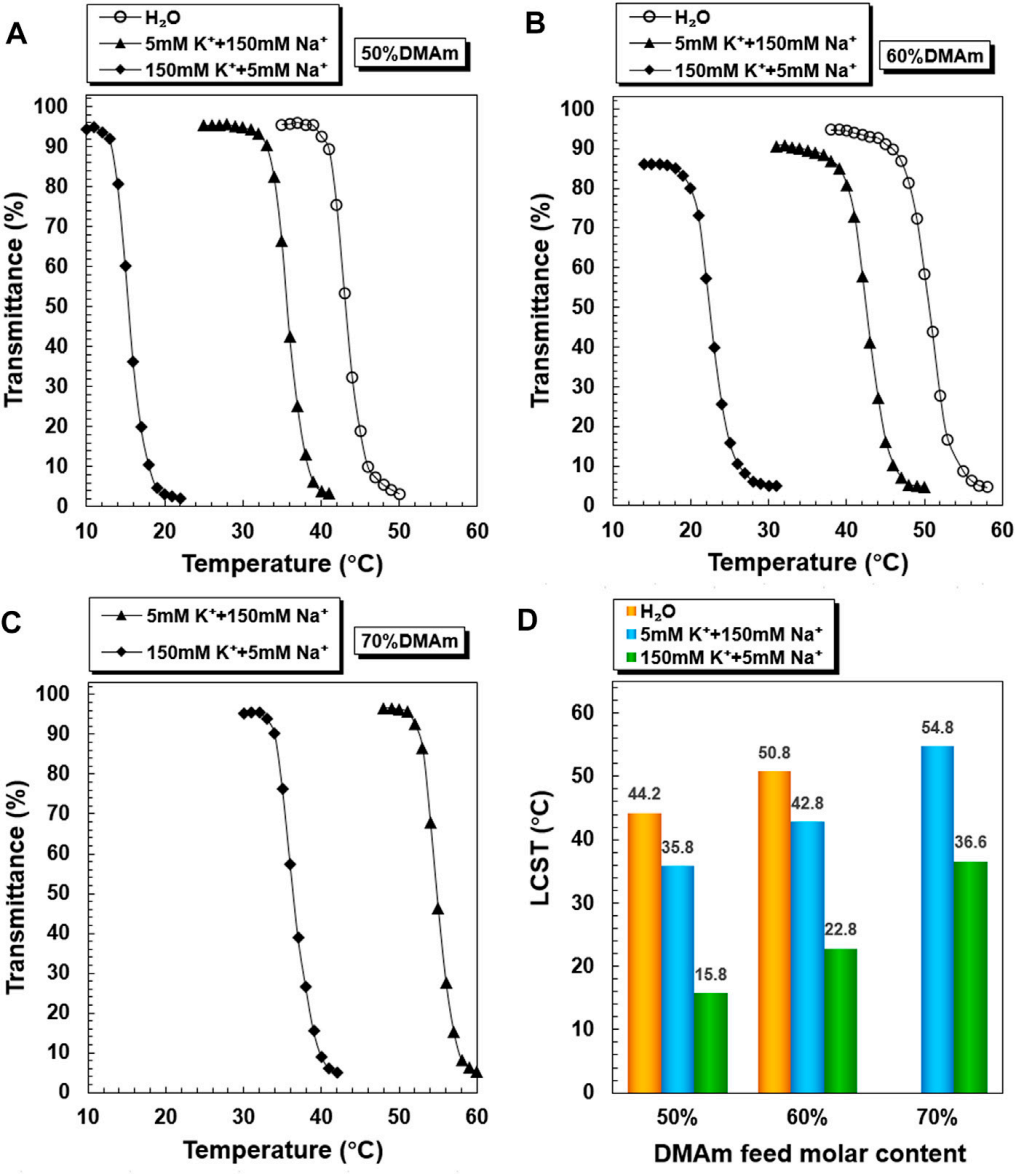
K^+^-responsive behaviors of PNDB-b-DSPE block copolymers. Temperature-dependent phase transition behaviors of PNDB-b-DSPE1 **(A)**, PNDB-b-DSPE2 **(B)**, and PNDB-b-DSPE3 **(C)** copolymer solutions with different K^+^ concentration levels. LCST values of PNDB-b-DSPE1, PNDB-b-DSPE2, and PNDB-b-DSPE3 copolymer solutions with different K^+^ concentration levels.

PNDB-b-DSPE1, PNDB-b-DSPE2, and PNDB-b-DSPE3 solutions with different K^+^ concentrations exhibit similar LCST shifts (Figure 3). The LCST values of PNDB-b-DSPE1, PNDB-b-DSPE2, and PNDB-b-DSPE3 block copolymers in simulated extracellular and intracellular fluids are almost the same as those of the corresponding PNDB1, PNDB2, and PNDB3 copolymers. That is, the combination of a DSPE block barely affects the K^+^-responsive behaviors of PNDB copolymers. When the ambient temperature is set at a certain temperature between the two LCSTs of PNDB-DSPE block copolymers in simulated extracellular and intracellular solution, block copolymers can change from a hydrophilic state to a hydrophobic state. In this study, normal physiological temperature (37°C) is chosen as the certain temperature. Herein, to achieve a successful intracellular drug release, the LCST values of PNDB-b-DSPE block copolymers need to be lower than 37°C in the simulated extracellular fluid and higher than 37°C in the simulated intracellular fluid. Therefore, PNDB-b-DSPE2 is chosen in this study to prepare micelles for subsequent experiments due to its appropriate LCST values in simulated intracellular and extracellular fluids and the large LCST shift values. In addition, the CMC value of PNDB-b-DSPE2 is 12 mg L^−1^ (Supplementary Figure S2).

### Characterization of Micelles

**FIGURE 4:**
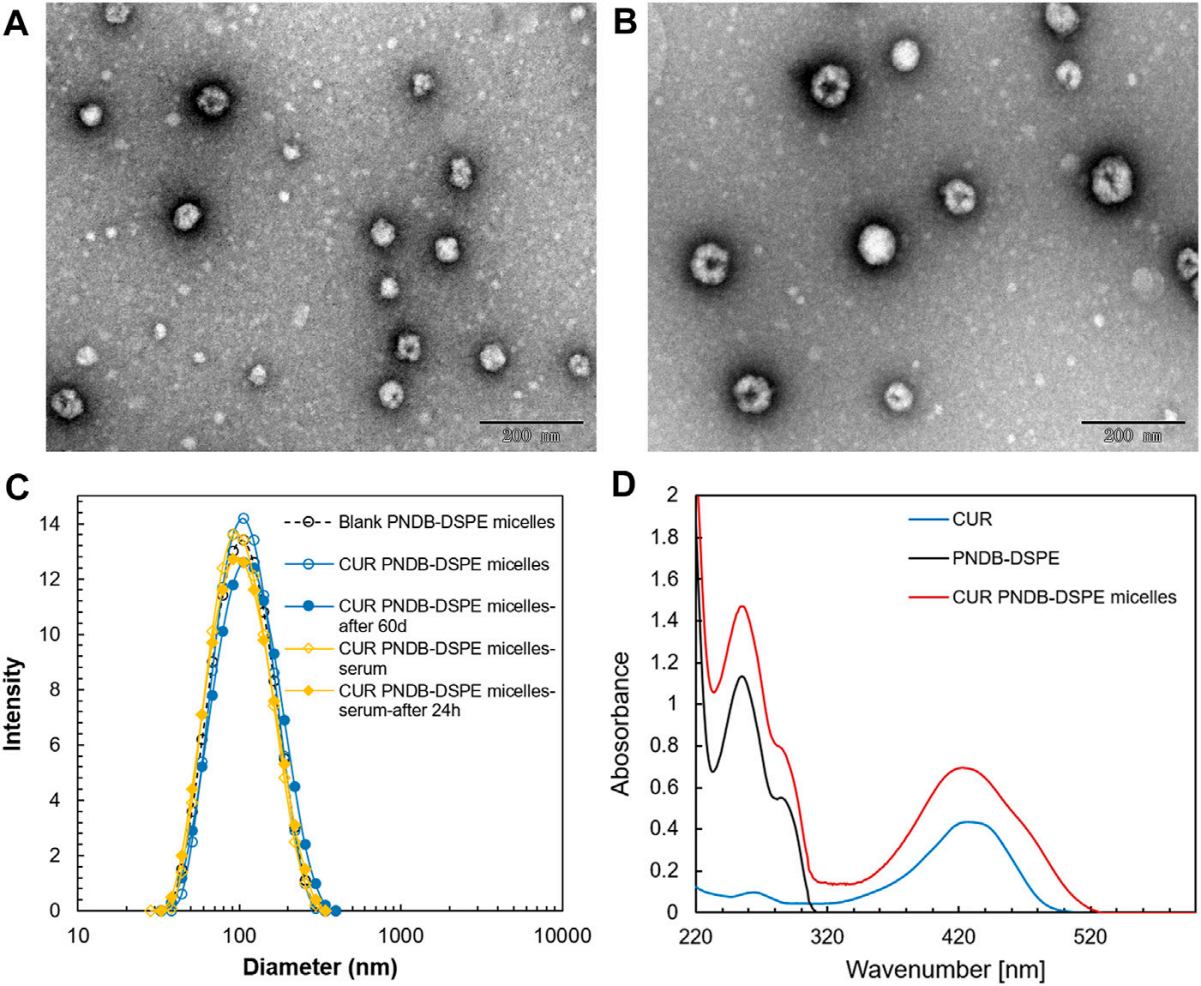
TEM images of blank PNDB-DSPE micelles **(A)** and CUR-PNDB-DSPE micelles **(B)**. Hydrodynamic size distributions of blank PNDB-DSPE micelles and CUR-PNDB-DSPE micelles **(C)**. The UV–Vis analysis of free CUR, blank PNDB-DSPE micelles, and CUR-PNDB-DSPE micelles **(D)**.

The prepared micelles are self-assembled by PNDB-b-DSPE2 copolymers and CUR. The morphology and size distribution of the micelles are characterized by TEM and DLS, respectively. As shown in Figure 4, blank PNDB-DSPE micelles and CUR-PNDB-DSPE micelles disperse well and exhibit good spherical shapes and fine monodispersity. The average diameters of dried blank PNDB-DSPE micelles and CUR-PNDB-DSPE micelles are about 60 and 80 nm, respectively. The hydrodynamic diameters of blank PNDB-DSPE micelles and CUR-PNDB-DSPE micelles are about 95 and 100 nm, respectively, with polydispersity index (PDI) values of 0.141 and 0.129, respectively. Moreover, their size distribution nearly remains the same even after 60 days in storage, indicating the good stability of the prepared micelles. Moreover, the hydrodynamic diameter of CUR-PNDB-DSPE micelles in serum is monitored, and significant variation of diameter after 24 h incubation in serum is not observed, which indicates that the stability of CUR-PNDB-DSPE micelles is not influenced by serum. The zeta potential of blank PNDB-DSPE micelles and CUR-PNDB-DSPE micelles is −4.84 and −11.73 mV, respectively. For the UV–Vis analysis, the absorption spectra of CUR-PNDB-DSPE micelles display absorption peaks at approximately 255 and 426 nm, which attribute to PNDB-b-DSPE copolymers and CUR, respectively, and thus confirm the successful synthesis of micelles. Moreover, CUR-loading content (LC) and encapsulation efficiency (EE) of CUR-loaded micelles are 6.62 and 58.27%, respectively.

### K^+^-Responsive Controlled-Release Behaviors of Micelles

**FIGURE 5:**
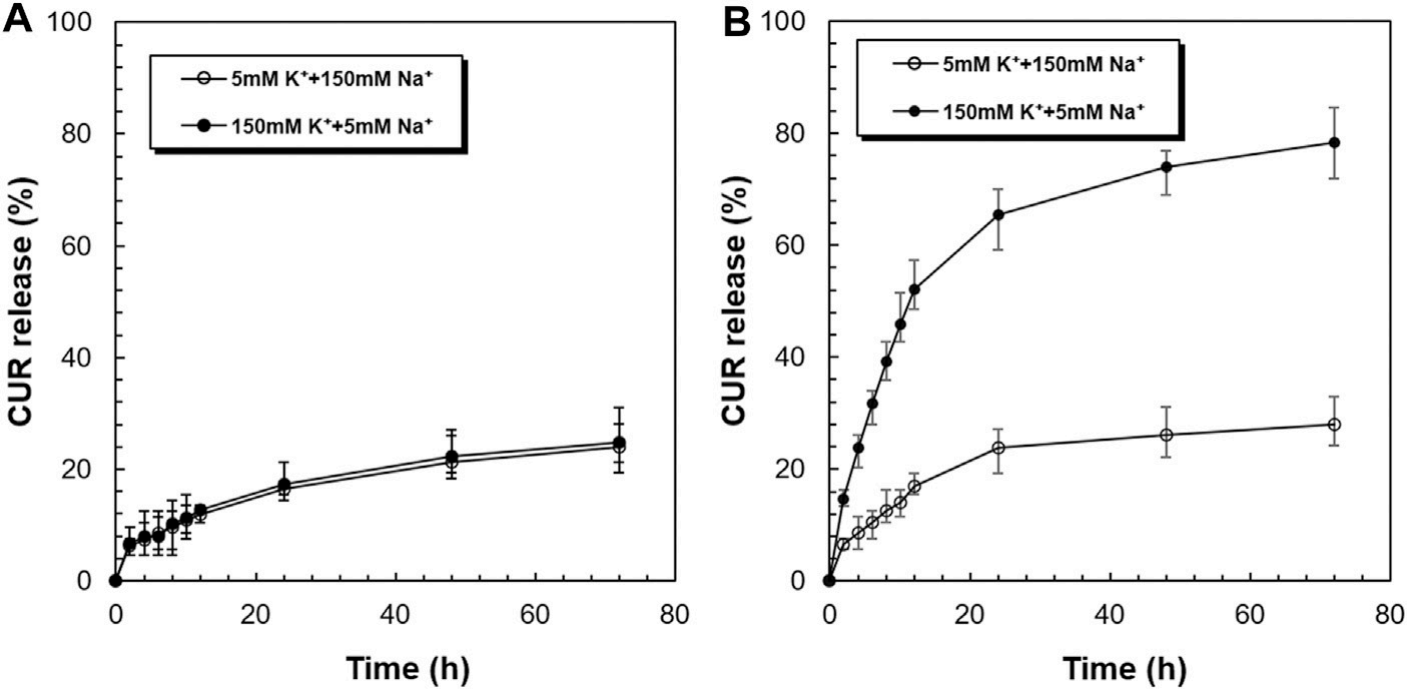
K^+^-responsive controlled-release behaviors of CUR from CUR-PEG-DSPE micelles **(A)** and CUR-PNDB-DSPE micelles **(B)** in simulated extracellular and intracellular fluids.

Figure 5 shows the *in vitro* release profiles of CUR from the CUR-PEG-DSPE micelles and CUR-PNDB-DSPE micelles at 37°C in simulated extracellular and intracellular fluids, respectively. As shown in Figure 5A, the release behaviors of CUR from CUR-PEG-DSPE micelles in simulated extracellular and intracellular fluids are almost the same, where both exhibit a burst release followed by a sustained slow release, reaching 23.75 and 24.87% at 37°C within 72 h, respectively. That is, the variation of K^+^ concentration nearly does not affect the drug release behaviors of CUR-PEG-DSPE micelles, which indicates that the CUR-PEG-DSPE micelles do not have K^+^-responsive controlled-release properties. In contrast, CUR-PNDB-DSPE micelles yield a much faster release in simulated intracellular fluids, while the same slow release is observed in simulated extracellular fluids and reaches 78.30 and 27.73% at 37°C within 72 h (Figure 5B). The results indicate that the drug release behavior of the CUR-PNDB-DSPE micelles is greatly affected by the environmental K^+^ concentration. Such K^+^-responsive controlled release properties of CUR-PNDB-DSPE micelles are achieved by altering the stability of the core-shell structure of micelles in response to the change in K^+^ concentration. The core-shell structure of CUR-PNDB-DSPE micelles could keep stable when the K^+^ concentration is about 5 mM, and CUR-PNDB-DSPE micelles demonstrate a slow drug release rate. When the K^+^ concentration is increased to 150 mM, the hydrophilic outer shell collapses due to the formation of 2 : 1 ‘‘sandwich’’ host–guest complexes of 15-crown-5 and K^+^, and the drug can migrate out of the micelles, and thus CUR-PNDB-DSPE micelles exhibit a fast release (Fleige et al., 2012).

Thus, CUR-PNDB-DSPE micelles are stable in simulated extracellular fluids while unstable in simulated intracellular fluids. Neither the early release of drug in a normal physiological environment nor the incapable drug release in the tumor sites can achieve a satisfactory therapeutic effect on solid tumors. Since the CUR-PNDB-DSPE micelles remain stable in a normal physiological environment and exhibit rapid drug release in intracellular fluid, these micelles are expected to be an appropriate candidate to deliver anticancer drugs for cancer therapy.

### Cellular Uptake and Intracellular Drug Release

**FIGURE 6:**
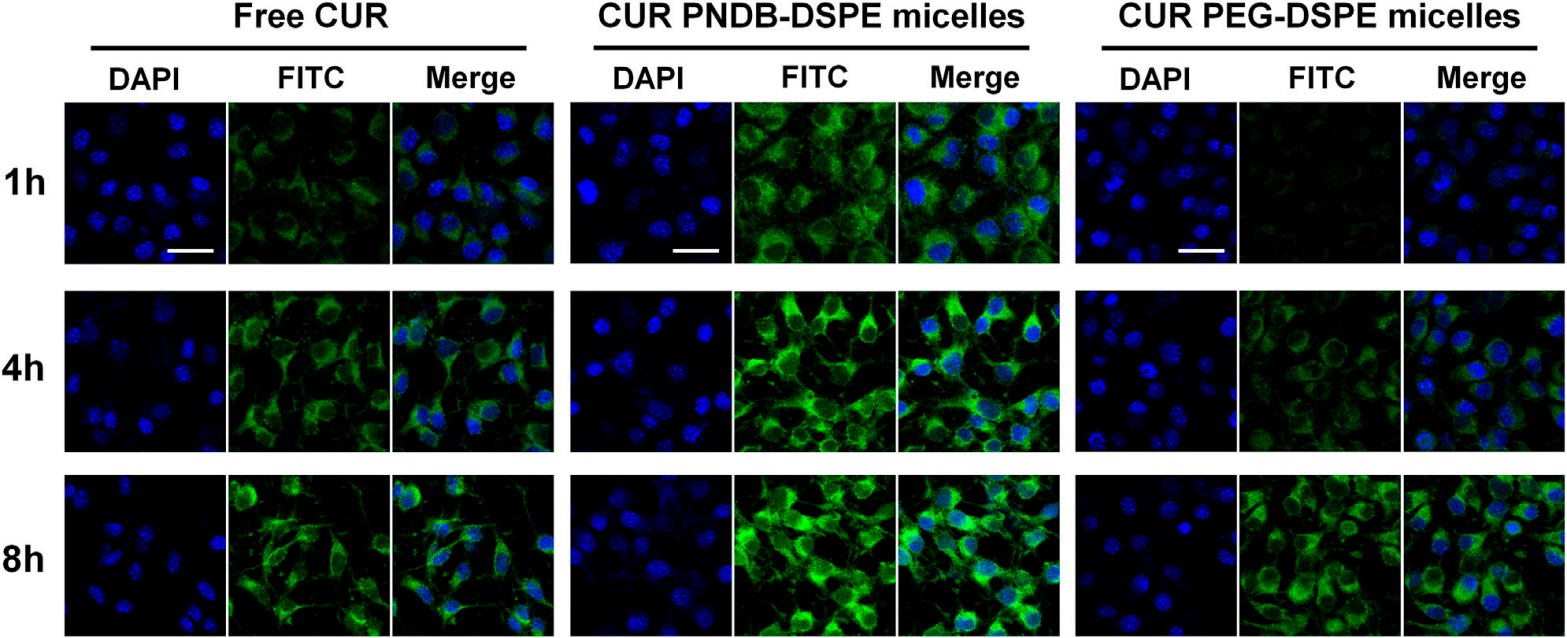
Confocal images of B16F10 cells incubated with free CUR, CUR-PEG-DSPE micelles, and CUR-PNDB-DSPE micelles for 1, 4, and 8 h at an equal CUR concentration of 20 μg mL^−1^; bars indicate 25 μm.

CUR has been reported to show anticancer activity against several types of tumors including melanoma, which is discussed herein, and B16F10 cells are used in further study. B16F10 cells are incubated with free CUR, CUR-PEG-DSPE micelles, and CUR-PNDB-DSPE micelles at an equal CUR concentration of 20 μg mL^−1^ for 1, 4, and 8 h, respectively, and then visualized under a fluorescence microscope to assess cellular uptake and intracellular drug release behaviors of the nanocarriers (Figure 6). The results demonstrate that these micelles are actively taken up by B16F10 cells as seen by green fluorescence within the cells. CUR-PNDB-DSPE micelles could rapidly accumulate in the cytosol of cells in 1 h revealed by bright green fluorescence compared to the cells treated with free CUR and CUR-PEG-DSPE micelles, which resulted in less intense fluorescence in the cytosol, indicating that CUR-PNDB-DSPE micelles exhibit a much higher cellular uptake. Furthermore, the intensity of green fluorescence in the cytosol after micelle treatment is much brighter after 4 h than in the 1 h treatment. Interestingly, comparing green fluorescence intensity in the cytosol after incubation for 8 h with 4 h, it is almost the same for cells treated with CUR-PNDB-DSPE micelles, while cells treated with free CUR and CUR-PEG-DSPE micelles all exhibit increased fluorescence intensity by increasing incubation time. The results demonstrate successful CUR release of CUR-PNDB-DSPE micelles due to the degradation of micelles in response to the high intracellular K^+^ concentration in the cells, and the release behaviors of CUR-PNDB-DSPE micelles can almost be completed within 4 h. Thus, the prepared CUR-PNDB-DSPE micelles have great potential as drug nanocarriers for excellent intracellular drug release in response to the intracellular K^+^ concentration.

### Cytotoxicity Assays

**FIGURE 7:**
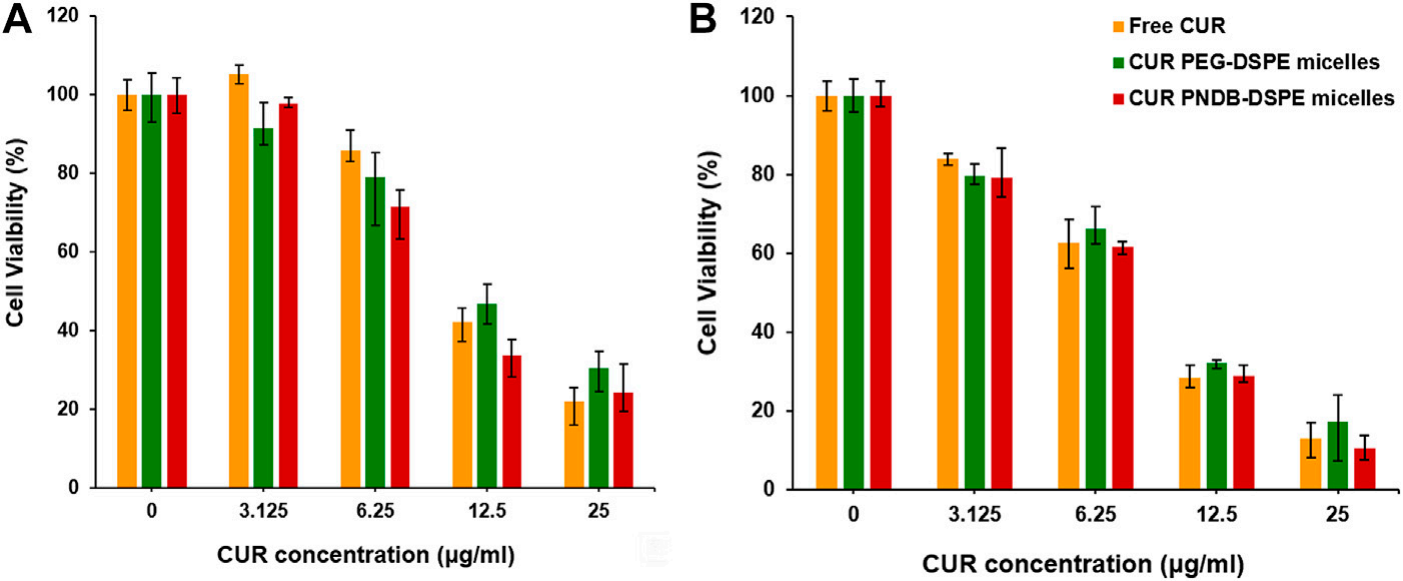
Cytotoxicity evaluation of B16F10 cells incubated with free CUR, CUR-PEG-DSPE micelles, and CUR-PNDB-DSPE micelles for 24 h **(A)** and 48 h **(B)** at different CUR concentration levels ranging from 3.125 to 20 μg mL^−1^.

To further evaluate the therapeutic efficacy of various CUR formulations, B16F10 cells are incubated with free CUR, CUR-PEG-DSPE micelles, and CUR-PNDB-DSPE micelles at CUR concentrations ranging from 3.125 to 25 μg mL^−1^ for 24 and 48 h. The results of cell viabilities are shown in Figure 7. After 24 h incubation, there is no significant cytotoxicity for free CUR and CUR formulations at low concentrations as 3.125 μg mL^−1^, and then the cell viability decreases with increasing CUR concentrations of free CUR and CUR formulations. When the concentrations exceed 6.25 μg mL^−1^, the toxicity of CUR-PNDB-DSPE micelles increases rapidly and is higher than that of CUR-PEG-DSPE micelles. When at the same CUR concentration, such as 12.5 μg mL^−1^, the toxicity of CUR-PNDB-DSPE micelles (33.6%) is significantly greater than that of CUR-PEG-DSPE micelles (46.7%). The results are consistent with the cellular uptake results. That is, CUR-PNDB-DSPE micelles could exhibit a high cellular uptake and excellent intracellular drug release in response to the intracellular K^+^ concentration and thus lead to increased cytotoxicity when compared to CUR-PEG-DSPE micelles. To verify whether the increased cytotoxicity of the CUR-PNDB-DSPE micelles is caused by the copolymers, a cytotoxicity assay of blank PNDB-DSPE micelles is performed, which shows good cell compatibility (Supplementary Figure S3). Moreover, free CUR also exhibits an obvious cytotoxicity property, which could be attributed to DMSO used to improve the solubility of CUR, which is toxic and could induce cell damage (Hanslick et al., 2009; Pal et al., 2011). Compared with incubation for 24 h, the cytotoxicity of free CUR and CUR formulations at various CUR concentrations is all increased after incubation for 48 h. For instance, when CUR concentration is 25 μg mL^−1^, the toxicity of free CUR, CUR-PEG-DSPE micelles, and CUR-PNDB-DSPE micelles after incubation for 24 and 48 h is 21.8%, 30.4%, and 24.3% and 12.8%, 17.3%, and 10.4%, respectively. The results indicate that CUR-PNDB-DSPE micelles show a high cytotoxicity to B16F10 cells and are expected to be an appropriate candidate for melanoma treatment.

## Conclusion

A novel type of polymeric micelles with intracellular K^+^-responsive controlled-release properties based on the responsive host–guest system has been successfully developed for delivering CUR. The polymeric micelles are self-assembled by PNDB-b-DSPE block copolymers and CUR, and the hydrophobic DSPE segments in the copolymers form the micellar core that acts as a reservoir for CUR, while the PNDB copolymers form the micellar shell with an obvious K^+^-responsive hydrophilic/hydrophobic phase transition behavior. The proposed CUR-PNDB-DSPE polymeric micelles exhibit good spherical shapes and fine monodispersity. When the environmental K^+^ concentration is significantly increased as the simulating intracellular fluid, the adjacent 15-crown-5 receptors in PNDB copolymers capture K^+^ to form stable 2 : 1 ‘‘sandwich-type’’ host–guest complexes, resulting in the hydrophilic outer shell to collapse and micelles to exhibit a fast release of CUR. CUR-PNDB-DSPE micelles demonstrate a high cellular uptake and excellent intracellular drug release in response to the intracellular K^+^ concentration of B16F10 cells. Moreover, CUR-PNDB-DSPE micelles show significant cytotoxicity to B16F10 cells compared to free CUR and CUR-PEG-DSPE micelles. The results in this study provide valuable guidance for designing and developing novel polymeric micelles with intracellular K^+^-responsive controlled release properties for delivering CUR for melanoma treatment.

## Data Availability

The original contributions presented in the study are included in the article/Supplementary Material; further inquiries can be directed to the corresponding author.
