# Supplementary material for: Intracellular K+-Responsive Block Copolymer Micelles for Targeted Drug Delivery of Curcumin
Source: Front Bioeng Biotechnol. 2022 Jun 30;10:919189. doi: 10.3389/fbioe.2022.919189 (PMC9280407; doi:10.3389/fbioe.2022.919189)
Supplement: Supplementary file 1 [file DataSheet1.docx]

Supplementary Material

Intracellular K^+^-Responsive Block Copolymer Micelles for Targeted Drug Delivery of Curcumin

Mingyue Jiang^12^, Le Chen^13^, Bo Chen^1^, Qinghua Yu^12^, Xianming Zhang^12^, Weihong Jing^12^, Limei Ma^13^, Tao Deng^12^, Zhangyou Yang^12^, Chao Yu^123*^

^1^ Chongqing Key Laboratory for Pharmaceutical Metabolism Research, College of Pharmacy, Chongqing Medical University, Chongqing, China

^2^ Research Center of Pharmaceutical Preparations and Nanomedicine, College of Pharmacy, Chongqing Medical University, Chongqing, China

^3^ Chongqing Pharmacodynamic Evaluation Engineering Technology Research Center, College of Pharmacy, Chongqing Medical University, Chongqing, China

*** Correspondence:**Corresponding Author
yuchao@cqmu.edu.cn (C Yu)


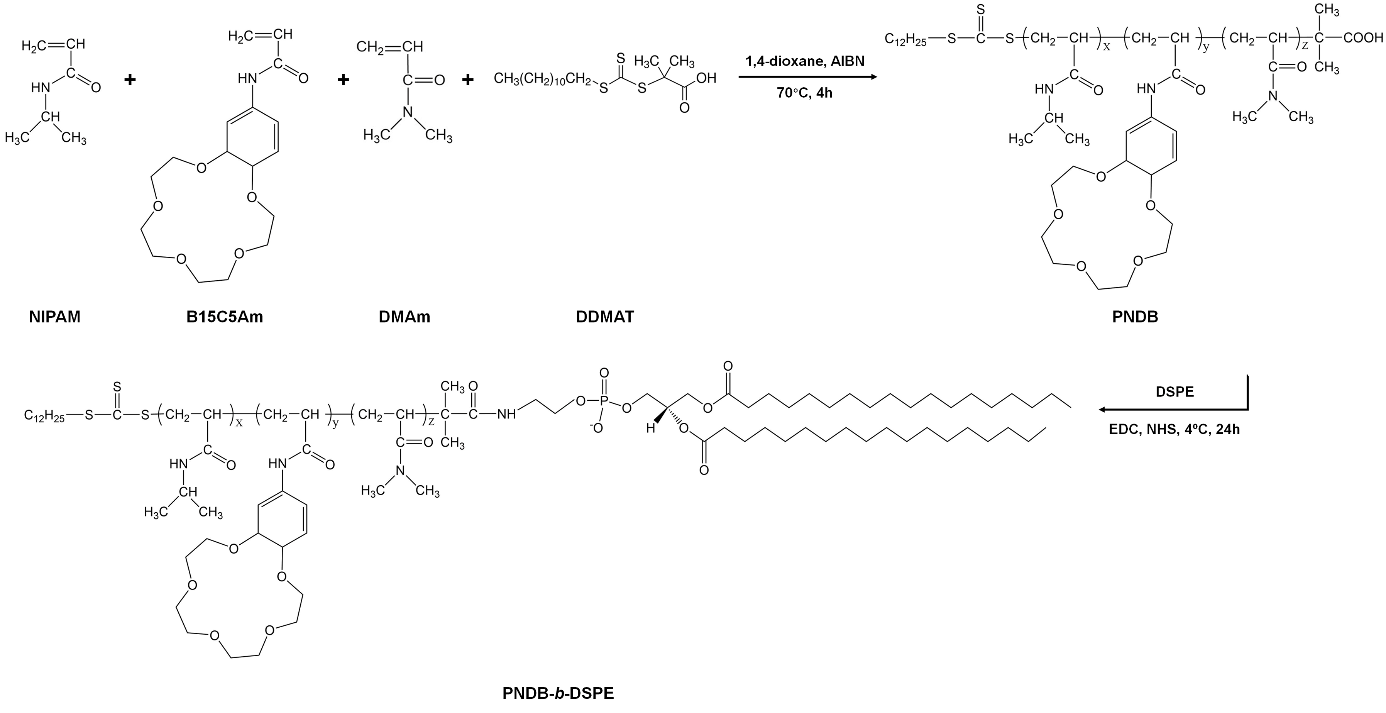


**Scheme S1.** Synthesis route of PNDB-b-DSPE.

**
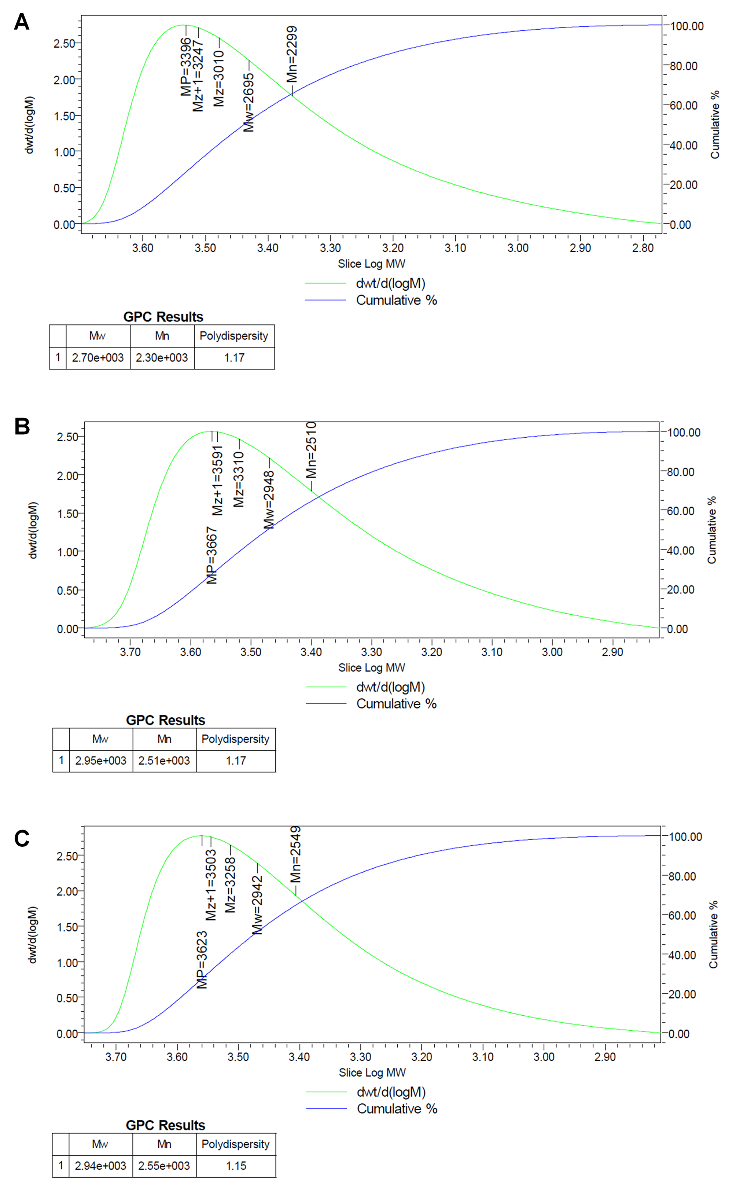
**

**Figure S1.** GPC of PNDB-b-DSPE 1 (A), PNDB-b-DSPE 2 (B) and PNDB-b-DSPE 3 (C) copolymers.


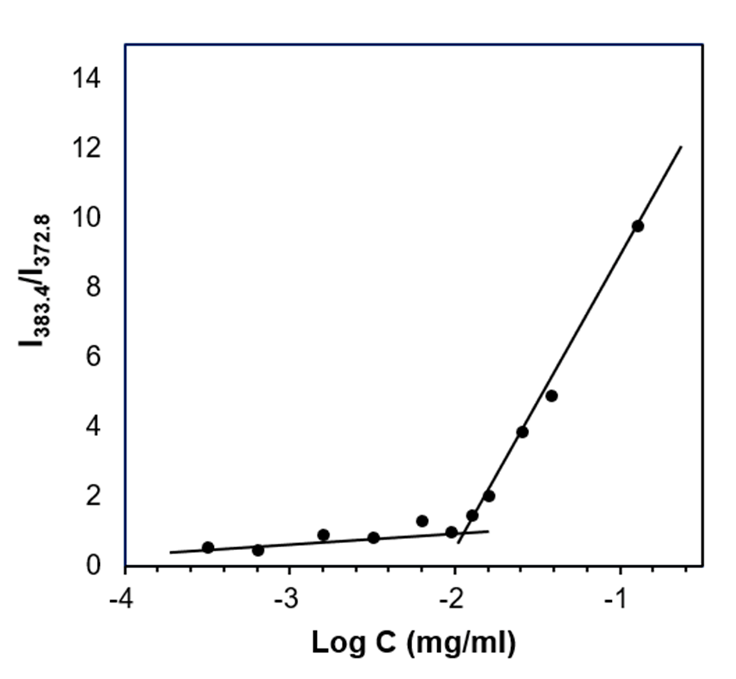


**Figure S2.** Plot of change of florescence intensity ratio (I_383.4_/I_372.8_) *versus* logarithm of

PNDB-b-DSPE2 copolymer concentrations.

**
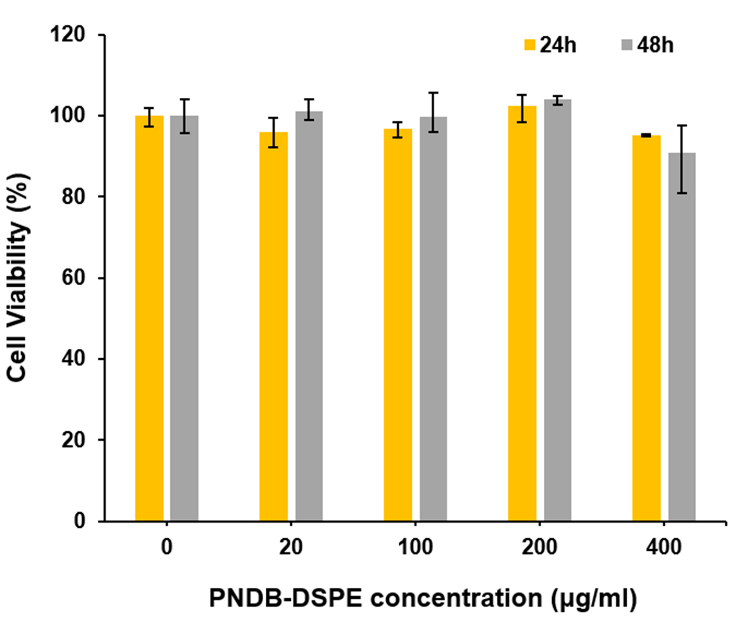
**

**Figure S3.** Cytotoxicity evaluation of B16F10 cells incubated with blank PNBD-DSPE micelles for 24 h and 48 h at different PNBD-DSPE concentrations ranging from 20 to 400 μg·mL^−1^.
